# Ursolic Acid, a Natural Nutraceutical Agent, Targets Caspase3 and Alleviates Inflammation‐Associated Downstream Signal Transduction

**DOI:** 10.1002/mnfr.201700332

**Published:** 2017-10-11

**Authors:** Xiaoyao Ma, Yuan Zhang, Zengyong Wang, Yunbing Shen, Man Zhang, Quandeng Nie, Yuanyuan Hou, Gang Bai

**Affiliations:** ^1^ State Key Laboratory of Medicinal Chemical Biology College of Pharmacy and Tianjin Key Laboratory of Molecular Drug Research Nankai University Tianjin China

**Keywords:** anti‐inflammation, CASP3, chemical biology, MAPK signaling pathways, ursolic acid

## Abstract

**Scope:**

Ursolic acid (UA) is a pentacyclicterpenoid carboxylic acid that is present in a wide variety of plant foods. There are many beneficial health effects that are attributed to the properties of UA. However, the specific cellular targets of UA and the mechanism underlying downstream signal transduction processes linked to the anti‐inflammation pathway have not been thoroughly elucidated to date.

**Methods and results:**

Chemical biology strategies such as target fishing, click reaction synthesis of a UA probe and molecular imaging were used to identify potential target proteins of UA. Cysteinyl aspartate specific proteinase 3 (CASP3) and its downstream signaling pathway were verified as potential targets by molecular docking, intracellular enzyme activity evaluation and accurate pathway analysis. The results indicated that UA acted on CASP3, ERK1 and JNK2 targets, alleviated inflammation‐associated downstream multiple signal transduction factors, including ERK1, NF‐κB and STAT3, and exhibited anti‐inflammation activities.

**Conclusion:**

As a natural dietary supplement, UA demonstrated anti‐inflammation activity via inhibition of CASP3 and shows the potential to improve the therapy effect of several inflammation‐associated diseases.

## 1 Introduction

There is growing interest in the health benefits associated with the consumption of phytochemicals from fruits, vegetables and herbal medicines.^1^ Plant‐derived products serve not only as dietary components but are also used to treat and prevent chronic diseases, such as obesity, diabetes, cardiovascular disorders, inflammatory and cancers.^2^ Pentacyclic triterpenoids, including oleanane, ursane and lupane groups, are widely distributed in many plants and are presumed to account for many of these beneficial effects.^3^, ^4^ Recently, ursolic acid (3β‐hydroxy‐urs‐12‐en‐28‐oic acid, UA), a pentacyclic terpenoid carboxylic acid, has been shown to regulate multiple proinflammatory transcription factors, cell cycle proteins, growth factors, kinases, cytokines, chemokines, adhesion molecules, and inflammatory enzymes.^5^ UA is also known to interact with multiple intracellular and extracellular protein targets, as well as playing a key role in apoptosis, metastasis, angiogenesis and inflammatory processes.^6^

It is noteworthy that clinical tests suggest the possibility of practical uses of UA for improving therapy for inflammatory diseases, including cancer,^7^, ^8^ or type 2 diabetes mellitus and associated complications.^9^ These results have led to increased awareness and attractiveness of UA and its ramification as the potential therapeutics. Zhang et al. summarized the anti‐tumor actions of UA,^10^ including anti‐cell DNA damage, anti‐tumor cell proliferation, anti‐angiogenesis, regulation of the immunological surveillance system, cell migration inhibition and invasion of tumor, and the inhibition of nuclear transcription factors to induce tumor cell apoptosis. Camer et al. demonstrated that UA can improve insulin signaling, reduce oxidative stress and inhibit proinflammatory signaling, which collectively imply an application to diabetes therapy.^11^ Although all the evidence has shown that UA is a potential candidate for the development of a comprehensive strategy for the treatment and prevention of health disorders,^12^ and the well‐investigated anti‐inflammatory, anti‐cancer effects and metabolic syndrome have been presented,^13^ there are few studies that have investigated the specific targets and molecular mechanism of UA.

As we know, the identification of the targets and mechanism of natural products is challenging and meaningful work. Given the rapid advances in chemical biology, effective chemical proteinomics methods have opened new avenues to probe compound‐target interactions. A Fishing‐rod strategy has been applied to verify that the target of indomethacin is GLO1 and that the anti‐inflammatory effects of the indomethacin were related to inhibition of GLO1 enzyme activity.^14^ Glycyrrhetic acid increased β2AR/Gas coupling and decreased receptor internalization through the azide‐modified GA probe employed co‐localization, which enhanced the efficacy of β2AR agonists through this novel mechanism and suggested a potential application for use in the prevention of tachyphylaxis.^15^ XPB/ERCC3 was confirmed as the key target of triptolide, which was shown to inhibit DNA‐dependent ATPase activity, suggesting triptolide is a new type of anti‐cancer agent.^16^ Thus, the identification of biological targets based on the physiological properties of natural products or drug‐based chemical probes has become a primary focus for investigators.

In this study, we employed chemical biology methods to prepare a UA probe used for target capture from BEAS‐2B cells. Subsequently, molecule docking, cell co‐localization and downstream signaling pathway evaluation were applied to verify the key target. The results of the biochemical analysis and molecular biological detection methods demonstrated that UA affects the action of cysteinyl aspartate specific proteinase 3 (CASP3) on the MAPK signaling pathway, regulates STAT3 and NF‐κB transcription, and alleviates the inflammation‐associated transduction process.

## 2 Experimental Section

### 2.1 Reagents

UA (purity >98.5%, determined by HPLC) and N, N‐Diisopropylethylamine (DIPEA) were purchased from Aladdin (Beijing, China). Sodium1‐((3‐((4‐azidophenyl) disulfanyl) propanoyl) oxy)‐2, 5‐dioxopyrrolidine‐3‐sulfonate (Sulfo‐SADP) was purchased from Bioworld (MN, USA). The synthesis of N_3_‐tag (3‐azido‐7‐hydroxy‐2H‐chromen‐2‐one) was delegated to Wuxi App Tec (Beijing, China). Fe_3_O_4_ amino magnetic microspheres (MMs) were purchased from Tianjin baseline Chromtech Research Center (Tianjin, China). Human TNF‐α was purchased from PeproTech (Rocky Hill, NJ, USA). The NF‐κB luciferase reporter plasmid pGL4.32 and Renilla luciferase reporter vector plasmid pRL‐TK were obtained from Promega (Madison, WI, USA). The transfection reagent Lipofectamine 2000 was obtained from Invitrogen (Carlsbad, CA, USA). Primary antibodies ERK1/2, JNK1/2, CASP3, NF‐κB (p65), STAT3, PARP, β‐actin, p‐STAT3^S727^, p‐ERK1/2, p‐JNK and secondary antibodies were purchased from Cell Signaling Technology (Beverly, MA, USA). Fluorescent‐labeled anti‐rabbit IgG secondary antibodies were purchased from Abcam (Cambridge, UK). Chemiluminescent HRP substrates were purchased from Millipore Corporation (MA, USA). CASP3 inhibitor Q‐VD‐Oph was purchased from Selleckchem (Texas, USA). All cell culture reagents were purchased from Gibco BRL Life Technologies (Grand Island, NY, USA). All other chemicals and solvents used were of analytical grade.

### 2.2 Cell Culture

BEAS‐2B cell line, derived from human bronchial epithelial cells, was cultured in Dulbecco's Modified Eagle's medium 1640 medium supplemented with 10% fetal bovine serum. Chinese hamster ovary (CHO) cells were cultured in Dulbecco's Modified Eagle's medium/F12 supplemented with 10% fetal bovine serum. All cells were obtained from the American Type Culture Collection (Manassas, VA, USA) and incubated at 37 °C in a 5% CO_2_ atmosphere in a humidified incubator. All cells were used at ≈75% confluency (cell density) in 6‐well plates or 25 cm^2^ culture flasks for western blot analysis and ≈60% confluency in 20 mm culture dish for immunocytochemical analysis.

### 2.3 Synthesis of Alkynyl‐Modified UA Probe

Oxalyl chloride (83 μL, 0.975 mmol) was added dropwise to a solution of UA (296.8 mg, 0.65 mmol) in 10 mL of anhydrous dichloromethane. The mixture was stirred under argon at room temperature for 6 h. Subsequently, the solvent was evaporated under reduced pressure to afford the crude intermediate as a white solid. This crude product and mono‐propargylamine (67 μL, 0.975 mmol) were then dissolved in 10 mL of anhydrous dichloromethane and stirred at room temperature for 4 h. The mixture was concentrated in vacuo and purified by column chromatography on silica gel (dichloromethane: methanol = 30:1) to obtain alkynyl‐modified UA probe as a white solid. The yield of this reaction was 56% (179.6 mg). The detailed NMR data are described in the supporting information.

### 2.4 Click Chemistry Reaction of UA Probe in Cells

BEAS‐2B cells were cultured in 20 mm culture dish with or without 10^−5^ mmol L^−1^ alkynyl‐modified UA probe for 6 h. BEAS‐2B cells were washed with PBS and fixed using 4% paraformaldehyde for 30 min and later treated with a 100 μL reaction solution (0.2 mmol L^−1^ N_3_‐tag, 0.2 mmol L^−1^ Tris‐triazoleamine, 1.0 mmol L^−1^ CuSO_4_, and 2.0 mmol L^−1^ sodium ascorbate in precooled PBS). After 1 h incubation, the cells were washed with PBST. The fluorescence images were obtained with a confocal microscope (Carl Zeiss, Oberkochen, Germany). The excitation and emission wavelengths employed were 365 and 470 nm, respectively.

### 2.5 Preparation of UA‐Modified Functionalized MMs

A total of 1 mL NH_2_‐MMs (5 mg mL^−1^) was suspended in 5 mL borate buffer and Sulfo‐SADP (0.5 mg, 11 μmol) was added; then the mixture was reacted at room temperature for 12 h. Subsequently, the azide modified‐MMs were enriched via magnetic separation and first washed three times with water followed by three washes with methanol, respectively. The azide modified‐MMs were collected and used directly for the subsequent steps. CuBr (10 mg, 0.7 mmol) was dissolved in degassed methanol (3 mL) under argon atmosphere and DIPEA (35 μL, 0.18 mmol) was added. The resulting blue suspension was degassed for 30 min under a stream of argon in the dark. Alkynyl‐modified UA (0.54 mg, 11 μmol) was dissolved in degassed methanol (0.5 mL) and treated with 1 mL of the freshly prepared suspension of CuBr‐DIPEA and azide modified‐MMs (25 mg, 5 mg mL^−1^). The reaction mixture was shaken at room temperature in the dark for 24 h. Next, the UA‐modified functionalized MMs were separated with a magnet and washed with methanol and water. The enriched MMs were reduced by DL‐ DTT (100 mmol L^−1^) and the released UA derivative was analyzed by LC‐MS (Shimadzu 2020, Japan). The details of UA‐modified functionalized MMs are shown in the supporting information.

### 2.6 Target Prediction of UA

The three‐dimensional (3D) structure of UA was put into the PharmMapper database (http://59.78.96.61/phammapper) for target prediction. The pathways were carried out through KEGG (http://bioinfo.capitalbio.com). The interaction of proteins was analyzed using String9.1 (http://www.string-db.org/). The 3D structures of potential protein targets were obtained from the Protein Data Bank (http://www.rcsb.org/pdb). The structure of the molecules and potential targets were constructed and minimized using the SYBYL software (Chemical Computing Group, Inc.), then, AutoDock version4.2 (Olson Laboratory, LaJolla, CA) was applied for the docking study using a hybrid Lamarckian genetic algorithm (LGA). The number of LGA runs was set to 30. The step size parameters of quaternion and torsion were 30. In addition, a Molecular Operating Environment (MOE 2015.10) was also used for molecular docking. The triangle matcher algorithm of the MOE software packages was selected to dock the identified compounds into the protein active site. The scoring function must comply with the following parameters: (i) specifying ASE Scoring to rank the poses output by the placement stage; (ii) specifying Forcefield Refinement to relax the poses; (iii) specifying Affinity dG Scoring to rank the poses using the refinement stage. The free energy of binding was calculated from the contributions of the hydrophobic, ionic, hydrogen bond, and van der Waals interactions between the protein and the ligand, intramolecular hydrogen bonds and strains of the ligand. We observed that the docking poses were ranked by the binding free energy calculation in the S field.

### 2.7 Targets Fishing

BEAS‐2B cells were plated in 25 cm^2^ culture flasks and stimulated by 20 ng mL^−1^ TNF‐α for 6 h. Subsequently, the cells were washed three times with precooled PBS. Next, the cells were cultured with 0.5 mL RIPA lysis buffer (China COSCO, Beijing, China) for 30 min at 4 °C and collected with a cell scraper. The lysate was centrifuged and the protein content of the supernatant was quantified with a BCA protein assay kit (Thermo Scientific, Waltham, MA, USA). The UA‐modified functionalized MMs were added to the lysate and incubated for 10 h at 4 °C. The MMs were collected by magnetic separation and rinsed three times with precooled PBS. Bound proteins were released with 200 μL DTT (100 mmol L^−1^) at 4 °C for 30 min and the supernatants were collected for SDS‐PAGE.

### 2.8 Co‐Localization of Target Protein and UA Probe

BEAS‐2B cells were cultured in 20 mm culture dish. When cells reached approximately 60% confluence, they were treated with 10^−5^mmol L^−1^ alkynyl‐UA probe for 6 h. After washing with PBS, the cells were fixed with 4% paraformaldehyde for 15 min, and washed with PBS. After blocking with 10% goat serum for 1 h, cells were incubated with rabbit against CASP3 polyclonal antibodies (1:1000) overnight at 4 °C. After washing with PBST, anti‐rabbit secondary cy3 antibodies (1:1000) were added for 1 h at room temperature. After washed, the fixed cells were carried to the click chemistry reaction of N_3_‐tag according to the previously described bioorthogonal conditions. All fluorescence images were obtained with a confocal microscope. The excitation and emission wavelengths of Cy3 employed were 590 and 617 nm, respectively.

### 2.9 Extracellular Protein Measurements of CASP3

After 20 ng mL^−1^ TNF‐α stimulation and UA treatment, the levels of activity of CASP3 in the cell lysates were measured using commercial CASP3 activity kits (Beyotime, Shanghai, China) according to the manufacturer's instructions. The absorbance values were determined at 405 nm using a BioTek ELx800 microplate reader (BioTek Instruments, Inc. USA). CASP3 activity was expressed as the fold change in enzyme activity compared to the control group.

### 2.10 NF‐κB (p65) and STAT3 Nucleus Translocation Assay

BEAS‐2B cells were grown in 20 mm culture dish for 24 h and pretreated with drugs (UA or Q‐VD‐Oph) for 6 h. Then the cells were treated with TNF‐α (20 ng mL^−1^) for 6 h, and subsequently carried for the observation of NF‐κB (p65) and STAT3 nucleus translocation. The cells were washed three times with PBST, fixed in 4% paraformaldehyde for 15 min, then permeabilized with 0.1% Triton X‐100 for 5 min. After the permeabilization, the samples were washed with PBST adequately, and blocked in 10% goat serum for 1 h at room temperature. The cells were then sequentially incubated with anti‐NF‐κB p65 antibody (1:400) or anti‐P‐STAT3^S727^ antibody (1:500) overnight at 4 °C. On the next day, the cells were probed with an Alexa Fluor 594‐conjugated anti‐rabbit antibody (1:500) at room temperature for 1 h, then washed with PBST adequately. Additionally, the DAPI Fluoromount‐G was added for 5 min to stain the nuclei. Images were captured with a confocal microscope (Carl Zeiss, Oberkochen, Germany).

### 2.11 SDS‐PAGE and Western Blot Analysis

Using a general protocol, 10% SDS‐PAGE was conducted. Briefly, captured protein was diluted with loading buffer, boiled, and loaded for SDS‐PAGE and Coomassie Blue staining. The same SDS‐PAGE gel was transferred to PVDF membranes for western blot analysis. After transfer, membranes were blocked with 5% non‐fat dry milk for 1 h and then incubated with primary antibody (1:1000) overnight at 4 °C. Membranes were rinsed with PBST three times, and then incubated with secondary antibody (1:1000) at room temperature for 1 h. Membranes were washed, incubated with chemiluminescent HRP substrates and exposed in a FluorChem E imager (ProteinSimple, Santa Clara, CA).

### 2.12 Statistical Analysis

The results are expressed as the mean ± standard deviation (SD). Multiple comparisons were performed using an analysis of variance (ANOVA), followed by Bonferroni's post hoc test. For single comparisons, significant differences between the means were determined using Student's *t*‐test. *p*< 0.05 was considered to indicate significant differences. All data were processed using the GraphPad Prism statistical software, version 5.01.

## 3 Results and Discussion

### 3.1 Molecular Imaging and Target Fishing in Cells

**Figure 1:**
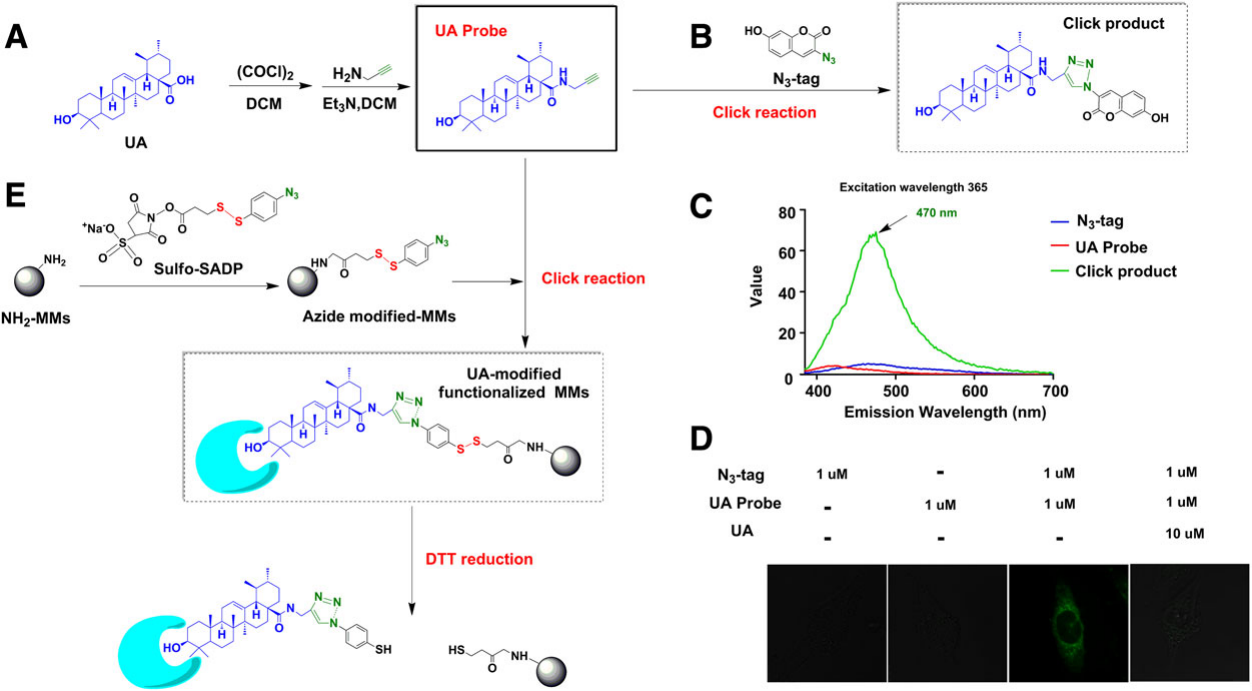
Synthesis and application of UA probe (A) Synthesis of alkynyl‐modified UA (UA probe); (B) Synthesis of the fluorescent click product; (C) Fluorescence intensity of the click product compared with the UA probe and N_3_‐tag; (D) Fluorescence imaging of UA probe and N3‐tag in BEAS‐2B cells; (E) Synthesis of UA‐modified functionalized MMs and the process of capture and release of the target protein.

To explore the exact target proteins of UA, an alkynyl‐modified UA probe was synthesized according to the scheme shown in **Figure** 1A. As shown in Figure 1B, equal amounts of the UA probe (group 1) and N_3_‐tag (group 2) were used to compare with the fluorescence intensity of the click product (UA‐N_3_‐tag, group 3). As expected, the click product showed strong fluorescence, while the two substrates (groups 1 and 2) showed almost no fluorescence. The excitation and emission wavelengths of the click product were 365 and 470 nm, respectively (Figure 1C). Under identical conditions, a fluorescence signal associated with the click product was observed in the cytoplasm of BEAS‐2B cells, and we demonstrated that the click product can be displaced from the cells by the addition of 10 μmol L^−1^ of free UA (Figure 1D). To identify the potential targets, the UA‐modified functionalized MMs were used to capture protein targets in TNF‐α‐stimulated BEAS‐2B cells. The captured proteins were released by DDT reduction and subsequently analyzed via SDS‐PAGE and western blotting (Figure 1E).

### 3.2 Target Prediction and Functional Analysis of UA

**Figure 2:**
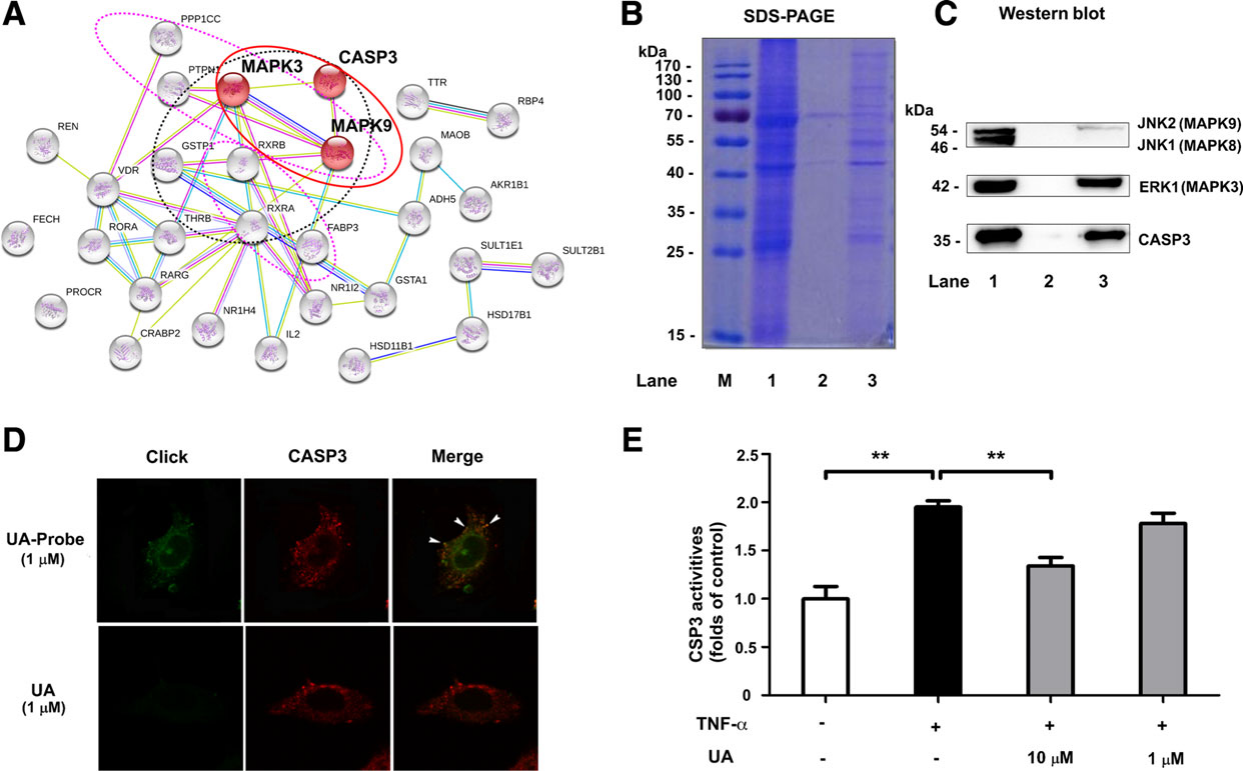
Prediction and verification of the targets of UA (A) PharmMapper and String 9.1 were used to predict and analyze the interaction and function of UA with target proteins; (B) SDS‐PAGE and (C) Western blot analysis were used to detect CASP3, ERK1 and JNK2 proteins enriched by UA‐modified functionalized MMs. Lane 1 was the total protein of the lysate of BEAS‐2B cells, lane 2 and lane 3 were the protein enriched from the lysate of BEAS‐2B cells by azide modified‐MMs and UA‐modified functionalized MMs, respectively; (D)The co‐localization of CASP3 (red) and UA probe (green) in BEAS‐2B cells; (E) Effects of UA on TNF‐α‐induced increase of CASP3 activity. The data represent mean ± SD of three groups; **p <0.01.

To screen the potential target proteins of UA, the top 30 protein targets (determined based on fit value using PharmMapper were software) were analyzed using bioinformatics tools. As shown in **Figure** 2A, there were three targets with anti‐inflammatory functions in the MAPK pathway, namely, MAPK9 (JNK2), MAPK3 (ERK1) and CASP3, all of which are described in the red balls. In addition, seven proteins with anti‐cancer functions (Black circles) and the other seven proteins with anti‐diabetes functions (Pink circles), respectively. These results were consistent with previous studies of UA activity.^13^ We then used AutoDock 4.0 software to conduct molecular docking studies to evaluate the interaction between anti‐inflammatory targets and UA. UA was found to target JNK2, ERK1 and CASP3 with ideal scores of –7.89, –10.36 and –8.82 kcal mol^−1^, respectively. Thus, these target proteins were selected for further verification by chemical proteomics.

### 3.3 Verification of Target Proteins

To evaluate the collecting efficiency, SDS‐PAGE was performed, and proteins were detected using Coomassie Brilliant Blue (Figure 2B). The results showed that some proteins were enriched from the lysate of BEAS‐2B cells by UA‐modified functionalized MMs (lane 3), but the intensity of the bands containing only azide modified‐MMs (lane 2, negative control group) was only slightly above the limit of detection. This result indicates that UA‐related proteins were selectively enriched and released by this fishing‐rod strategy. To further verify these results, the same SDS‐PAGE gel was transferred to PVDF membranes for western blot analysis and western blots were performed to determine the most likely targets of JNK2, ERK1 and CASP3 enrichment. As shown in Figure 2C, the bands of ERK1 and CASP3 proteins were clearly enriched compared to the negative control group. However, capture of JNK2 by UA‐modified functionalized MMs was comparatively low. The results indicated that CASP3 and ERK1 were the primary target proteins of UA within the MAPK signaling pathway.

To verify the result of the above fishing‐rod strategy, the co‐localization of CASP3 and UA probe was investigated. As shown in Figure 2D, molecular imaging showed that the green fluorescence of UA probe was clearly observed only in the cytoplasm. Unlabeled UA showed little fluorescence in control group. The distribution of CASP3 (stained red by cy3 antibodies) was observed in cytoplasm and preferably associated with the UA probe (Green). These results of the chemical imaging suggest that the UA probe appears to partially co‐localize with CASP3 proteins within the cellular context (Yellow). To assess the inhibitory effects of UA, activation of CASP3 in BEAS‐2B cells was measured sequentially. As shown in Figure 2E, the activity of CASP3 was increased greatly upon TNF‐α stimulation, and decreased following the addition of 10 μmol L^−1^ UA.

### 3.4 Molecular Docking of UA with CASP3

**Figure 3:**
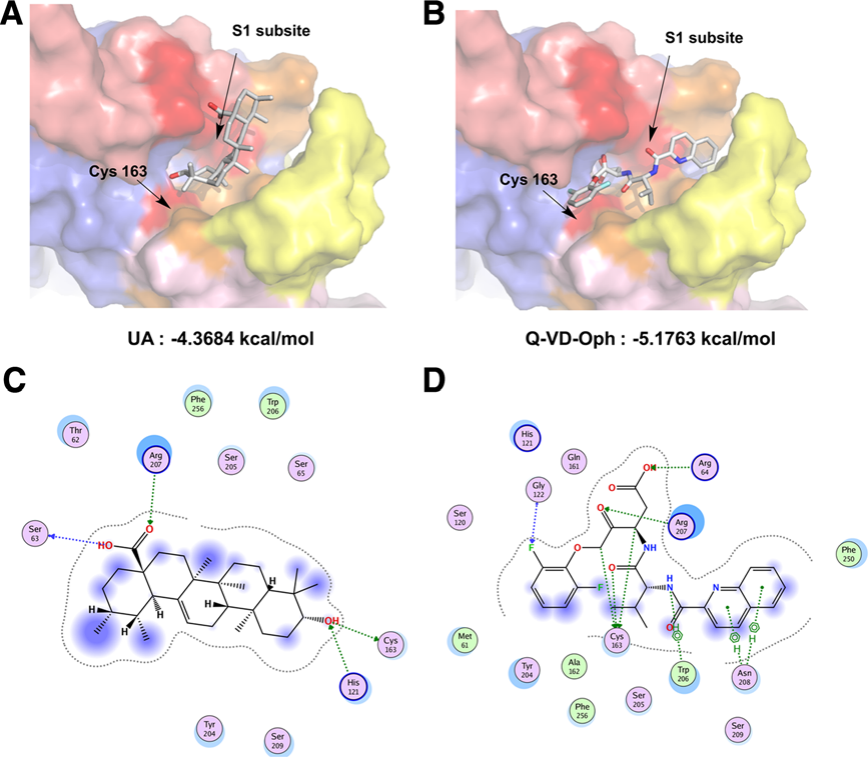
Molecular modeling of CASP3 and UA. Pymol software was used to display the 3D maps of the interaction of CASP3 (PDB: 5IC4) with UA (A) and Q‐VD‐Oph (B). UA and Q‐VD‐Oph are displayed as sticks and colored by atom type, with carbon atoms in gray. The S1 subsite and Cys‐163 that interact with UA or Q‐VD‐Oph are shown in red. The 2D depiction of protein‐ligand interaction between CASP3 and UA (C) and Q‐VD‐Oph (D). The interaction of residues and ligand are shown as green lines.

To provide additional insights on the interaction of UA and CASP3, we docked both UA and the inhibitor Q‐VD‐Oph to the binding site of CASP3 (PDB: 5IC4) using MOE software. We found that the binding energy of UA (–4.3684 kcal mol^−1^) was lower than that of Q‐VD‐Oph (–5.1763 kcal mol^−1^), and these findings were verified by the results of an enzyme activity detection kit. This technique indicated that hybrid Lamarckian genetic algorithm has a high probability to generate realistic‐binding poses. We then analyzed the top scoring poses of UA in all cases, which are displayed as 3D maps (**Figure** 3A and B) and 2D maps (Figure 3C and D) indicating the interaction of UA or Q‐VD‐Oph with CASP3. The carboxyl group of UA established hydrogen bonds with the active site amino acids Arg‐207 and Ser‐63, which is the same as Q‐VD‐Oph acting on the S1 subsite.^17^ Meanwhile, UA may interact with Cys‐163 and His‐121 via a previously proposed Cys‐His catalytic dyad hydrolysis mechanism, wherein the side‐chain of His‐121 approaches Cys‐163 to participate in the catalytic reaction.^18^ Q‐VD‐Oph has a similar impact on Cys‐163, which is the catalytic site of CASP3 (Figure 3C and D).

### 3.5 UA Inhibits the Action of CASP3

**Figure 4:**
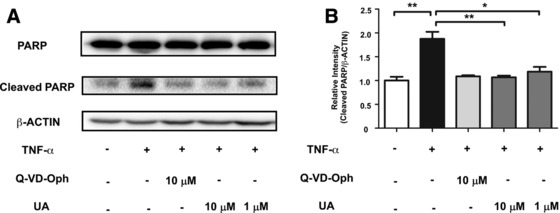
UA attenuated TNF‐α‐induced CASP3 activation and proteolytic processing of PARP (A) CHO cells were stimulated by 20 ng mL^−1^ TNF‐α for 12 h with or without 6 h pretreatment with UA or Q‐VD‐Oph. The cleaved PARP was measured by Western blot analysis. (B) The relative intensity data of cleaved PARP to β‐actin represent mean ± SD of three group; *p < 0.05 and **p <0.01.

Others have shown that the key feature of CASP3 in the cell is that they exist as zymogens, which are inactive until activated in the apoptotic cell both by extrinsic and intrinsic pathways.^19^ After undergoing proteolytic processing, the active heterotetramer of CASP3 can then hydrolyze the substrate protein to produce two subunits. Poly ADP‐ribose polymerase (PARP), a nuclear transcription protein that serves as the substrate of CASP3, plays an essential role in the regulation of cellular differentiation, development and metabolism.^20^ In this work, the fragmentation process of PARP was demonstrated, and it was used to evaluate the function of UA via inhibition of CASP3. As shown in **Figure** 4, the cleaved fraction of PARP in cells was significantly increased in TNF‐α induced model group. However, after treatment with UA or Q‐VD‐Oph, the fraction of cleaved PARP was significantly reduced. These results indicate that UA attenuated TNF‐α‐induced CASP3 activation and alleviated proteolytic processing of PARP in CHO cells.

### 3.6 UA Inhibits the Signal Transduction of Downstream Inflammatory Molecules

**Figure 5:**
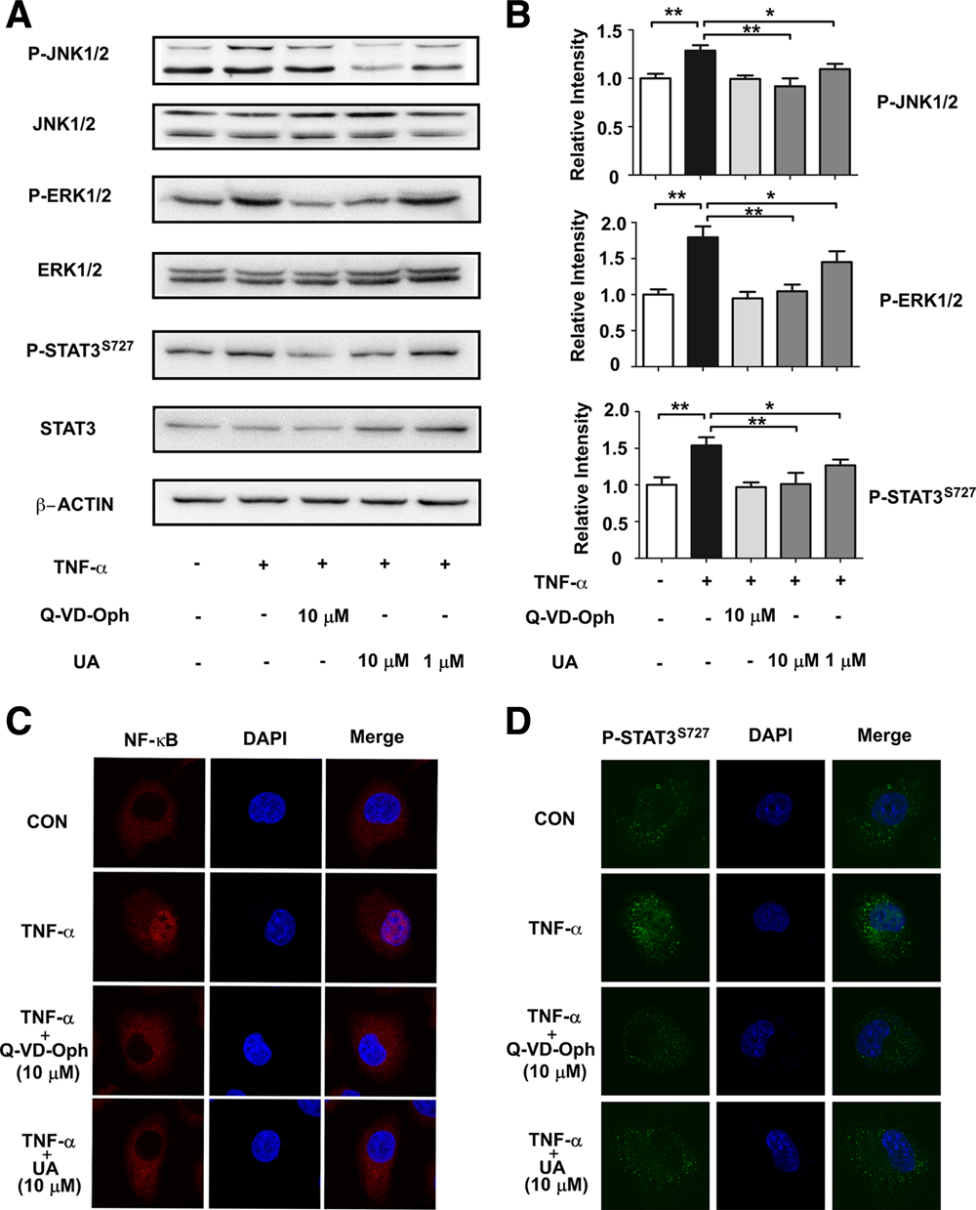
UA inhibits the signal transduction of downstream inflammatory molecules. (A)UA and Q‐VD‐Oph attenuated 20 ng mL^−1^ TNF‐α‐induced phosphorylation of ERK1/2, JNK1/2 and STAT3^S727^. (B) The relative intensity data of P‐JNK1/2, P‐ERK1/2 and P‐STAT3^S727^ to β‐actin represent mean ± SD of three group; *p < 0.05 and **p <0.01. UA and Q‐VD‐Oph attenuated TNF‐α‐induced unclear translocation of NF‐κB (C) and P‐STAT3^S727^ (D) in BEAS‐2B cells.

To confirm the molecular mechanisms underlying the anti‐inflammatory effects of UA in the downstream pathway of CASP3, we examined the inhibitory effects of ERK1/2, JNK1/2, NF‐κB, as well as STAT3 phosphorylation, all of which are mediated by CASP3. Western blot analysis revealed that the phosphorylation of ERK1/2, JNK1/2 and STAT3^S727^ were increased in TNF‐α induced group and markedly inhibited by UA and Q‐VD‐Oph in a concentration‐dependent manner (**Figure** 5A). We next investigated the inhibitory effect on the CASP3‐mediated nuclear translocation of NF‐κB and P‐STAT3^S727^.^21^ As shown in Figure 5B and C, TNF‐α stimulation caused the translocation of NF‐κB (p65) and P‐STAT3^S727^ from the cytosol to the nucleus. However, the translocation was significantly reduced by UA or Q‐VD‐Oph treatment. Taken together, these results suggest that UA alleviates inflammation by target CASP3 and impacts downstream molecule transduction.

## 4 Discussion

Inflammation is a double‐edged sword. On one hand, inflammation can provide protection to the human body by enclosing injury, destroying invaded bacteria and restoring the tissue or organs for recovery.^22^ On the other hand, excessive (chronic) inflammation has been linked with various chronic diseases such as obesity, diabetes, cardiovascular disorders and some cancers.^23^ A previous study reported that UA can protect mouse liver from CCl_4_‐induced inflammatory response by decreasing the activation of JNK, p38 MAPK and ERK in the MAPK signaling pathway and by regulating the NF‐κB signaling pathway.^24^ Moreover, UA was able to inhibit mitogen‐induced phosphorylation of ERK and JNK as well as their upstream kinases, MEK and c‐Raf, in lymphocytes.^25^ These studies suggest UA affects inflammation by suppressing target proteins in MAPK, NF‐κB and JAK‐STAT signaling pathways, although the specific targets were not identified. In the present study, CASP3 was identified as a target of UA and it was verified by utilizing a series of chemical biology methods. In the process, an alkynyl‐modified UA probe played a crucial role. Alkynyl‐modified UA probe exhibited favorable potential for target capture and visual imaging of cellular localization. The modification of alkynylation did not affect activity relative to free UA, resulting in a similar inhibition of expression of NF‐κB (in supporting data).

To the best of our knowledge, we show for the first time that the pentacyclic terpenoid UA exhibits an inhibitory effect on CASP3. As a cysteinyl aspartate‐specific proteinase, CASP3 shares many typical characteristics common to the cysteine‐aspartic acid protease family. For example, its active site contains a cysteine residue (Cys‐163) and a histidine residue (His‐121) that act to stabilize the peptide bond cleavage of the target protein sequence to the carboxy‐terminal side of an aspartic acid when it is part of a particular 4‐amino acid sequence (Asp‐x‐x‐Asp).^26^ Hence, molecular docking shows that the hydroxyl group at the C‐3 site of UA participates in a hydrogen bonding interaction with Cys‐163 and His‐121 (Figure 3C), and inhibits the hydrolysis of the substrate protein containing cysteinyl aspartate residues. This indicates that UA has a different action mechanism compared with conventional peptide inhibitors and some non‐peptidic inhibitors. Among the existing non‐peptidic inhibitors, such as (S)‐(+)‐5‐[1‐(2‐methoxymethyl‐pyrrolidine) sulfonyl] isatin and other isatin sulfonamide analogues, its C‐3 atom forms a covalent bond with the thiols of Cys‐163 and iron(III) chelating agent desferoxamine which forms an iron–sulfur complex with the thiols of Cys‐163;^27^, ^28^ in conclusion, UA is a novel natural CASP3 inhibitor.

**Figure 6:**
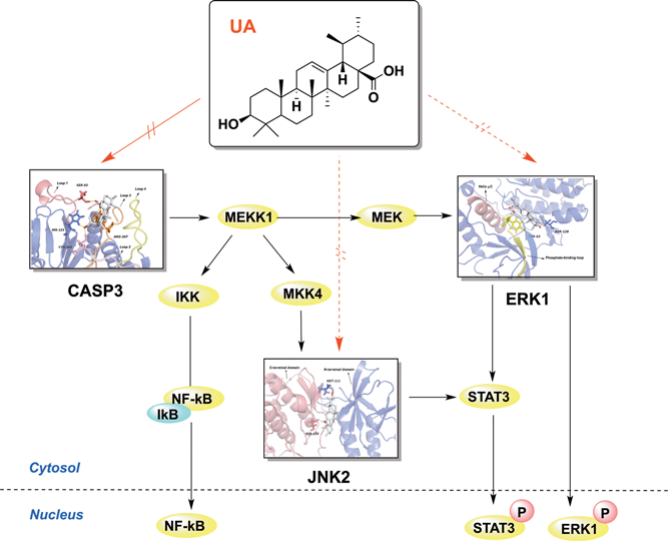
Effects of UA on the anti‐inflammatory response are hypothesized to be mediated by the MAPK signaling pathways.

As we know, CASP3 can cleave MEKK1, then active Raf, and subsequent MAPK pathway involved in the signaling pathways that are associated with inflammation.^29^ This is consistent with a previous report that CASP3 induces ERK1/2 activation through a ceramide‐dependent protease activity‐independent mechanism.^30^ TNF‐α or IL‐1β activate CASP3, which triggers downstream targets and the transcription of inflammatory genes, resulting in the release of such cytokines as NF‐κB, IL‐6, and NO.^31^ Moreover, activated ERK1 is translocated to the nucleus where it phosphorylates nuclear targets and leads to the release of cytokines. Others have shown that UA not only inhibits the expression of IL‐6‐induced STAT3 but also downregulates STAT3 by regulating gene products, such as cyclin D1, Bcl‐2, Bcl‐xL, survivin, Mcl‐1 and VEGF.^10^ Pathak et al. clearly highlighted the potential of UA to modulate the STAT3 signaling cascade in multiple myeloma. These researchers found that UA can inhibit both constitutive and IL‐6‐inducible STAT3 activation that correlated with the suppression of activation of upstream kinases (c‐Src, JAK1/2, and ERK1/2).^32^ This report supports our findings that UA targets CASP3, ERK1 and JNK2, and subsequently suppresses the activation of downstream ERK1, NF‐κB and STAT3, reduces the expression of cytokines and alleviates inflammation disorders (**Figure** 6).

Currently, there is a concern surrounding the use of natural compounds or products as daily dietary nutrition to prevent chronic inflammatory diseases. Moreover, daily supplements of UA have been shown to be safe and lead to a reduced risk of chronic inflammatory diseases. UA improves therapies for inflammation and inflammatory diseases, including cancer, type 2 diabetes mellitus and associated complications. Our findings suggest that the anti‐inflammatory effect of UA is due to the reduced expression of cytokines and alleviation inflammatory disorders via inhibition of CASP3. However, the targets of other effects, including improved type 2 diabetes mellitus, were not found in this study, and the identification of these targets requires further study. In summary, these results provide an increased understanding of UA, particularly regarding the molecular mechanism of anti‐inflammation as well as demonstrating the potential of UA as a therapeutic for the treatment of inflammation‐associated diseases.

**Abbreviations**
CASP3: cysteinyl aspartate specific proteinase 3
DIPEA, N: N‐Diisopropylethylamine
MMs: magnetic microspheres
PARP: poly ADP‐ribose polymerase
UA: ursolic acid

## Conflict of Interest

The authors have declared no conflicts of interest.

## Supporting information

Supporting Information
