# Supplementary material for: Ursolic Acid, a Natural Nutraceutical Agent, Targets Caspase3 and Alleviates Inflammation‐Associated Downstream Signal Transduction
Source: Mol Nutr Food Res. 2017 Oct 11;61(12):1700332. doi: 10.1002/mnfr.201700332 (PMC5765441; doi:10.1002/mnfr.201700332)
Supplement: Supplementary file 1 — Supporting Information [file MNFR-61-na-s001.docx]

**Supporting information**

1. **Synthesis section**

**General Chemical Reagents and Methods**

All purchased reagents for synthesis were used without further purification. All solventswere available commercially, dried or freshly dried and distilledprior to use. Thin-layer chromatography (TLC) was performed on silica gel GF254 plates with detection using shortwaveUV light (λ=254 nm) and staining with 10% phosphomolybdic acid in EtOH, followed by heating on a hotplate. Flash chromatography was performed with silica gel (100-200 mesh) with EtOAc/ petroleum ether or CH_2_Cl_2_/ MeOH as eluent. ^1^H and ^13^C NMR spectra were recorded on a Bruker AV 400 spectrometer at 400 MHz (^1^H NMR) and 100 MHz (^13^C NMR), using CDCl_3_ as solvents. Coupling constants are reported in Hertz.

**Figure S1**. Synthetic route for compound 3 (alkynyl-modified UA probe). Reagents and conditions: (a) oxalyl chloride，dichloromethane; (b) mono-Propargylamine, dichloromethane.

**Compound 3 Synthesis (****alkynyl-modified UA probe)**

To a solution of **compound 1** (296.8 mg, 0.65 mmol) in 10mL anhydrous dichloromethane, oxalyl chloride (83 µL, 0.975 mmol) was added. The mixture was stirred under argon at room temperature for 6 h. After that, the solvent was evaporated under reduced pressure to afford the crude intermediate **compound 2** as a white solid. The crude product above and mono-propargylamine (67 µL, 0.975 mmol) were dissolved in 10 mL anhydrous dichloromethaneand stirred at room temperature for 4 h. The mixture was concentrated in vacuo and purified by column chromatography on silica gel (dichloromethane: methanol = 30:1) to get the desired products **compound 3**, a white solid in 56% yield (179.6 mg). ^1^H NMR (400 MHz, CDCl_3_) δ 6.07 (t, *J* = 4.0 Hz, 1H), 5.36 (s, 1H), 4.03 (ddd, *J* = 17.5, 5.0, 2.3 Hz, 1H), 3.93 – 3.84 (m, 1H), 3.22 (dd, *J* = 11.0, 4.5 Hz, 1H), 2.20 (s, 1H), 2.01 – 1.94 (m, 3H), 1.87 (dd, *J* = 16.2, 7.0 Hz, 2H), 1.73 (d, *J* = 13.6 Hz, 1H), 1.63 (dd, *J* = 17.7, 7.0 Hz, 4H), 1.49 (ddd, *J* = 16.4, 14.1, 8.0 Hz, 9H), 1.39 – 1.22 (m, 6H), 1.10 (s, 3H), 0.99 (s, 3H), 0.95 (s, 4H), 0.93 (s, 3H), 0.87 (d, *J* = 6.4 Hz, 3H), 0.80 (s, 3H), 0.78 (s, 3H). ^13^C NMR (100 MHz, CDCl_3_) δ 178.05, 139.94, 126.23, 79.13, 77.48, 77.16, 76.84, 71.71, 55.31, 53.92, 47.93, 47.71, 42.62, 39.88, 39.73, 39.22, 38.92, 38.83, 37.09, 37.04, 32.87, 30.99, 29.52, 28.27, 27.99, 27.36, 25.12, 23.56, 23.43, 21.34, 18.42, 17.36, 17.08, 15.74, 15.66 (Figure S2)..

**Figure S2**. The NMR data of alkynyl-modified UA probe, (A) ^1^H NMR spectrum of alkynyl-modified UA probe (400 MHz, CDCl_3_) and (B) ^13^C NMR spectrum of alkynyl-modified UA probe (100 MHz, CDCl_3_).

1. **UA-modified functionalized MMs characterization section**

Fe_3_O_4_ amino magnetic microspheres (NH_2_-MMs) were purchased from Tianjin baseline chromtech research centre. The synthetic route for UA-modified functionalized MMs was shown in Figure S3. The release of UAprobe from UA-modified functionalized MMs was characterized with LC-MS-2020 (Shimadzu, Japan).

**Figure S3**. Synthetic route for UA-modified functionalized MMs (compound 6). Reagents and conditions: (a) MMs-NH_2_, borate buffer, DMSO; (b) CuBr, DIPEA, methanol ; (c) DTT, methanol.

**Azide modified-MMs Synthesis (Compound 5)**

5 mL NH_2_-MMs (25 mg, 5 mg/mL) were suspended in 2 mL borate buffer andSulfo-SADP (compound 4) (0.5 mg, 11 μmol) were added in sequence, the mixture was shocked at room temperature for 12 h. After that, the azide modified-MMs was enriched through magnetic separation and washed with water for three times. The gathered azide modified-MMs **(Compound 5)**was used directly for next steps.

**UA-modified functionalized MMs (Compound 6)**

CuBr (10 mg, 0.7 mmol) was dissolved in degassed methanol (3 mL) under argon atmosphere and DIPEA (35 μL, 0.18 mmol) was added. The resulting yellowish suspension was degassed for 30 min under a stream of argon and with exclusion of light. Alkynyl-modified UA probe **compound 3** (0.54 mg, 11 μmol) was dissolved in degassed methanol (0.5 mL) and treated with 1 mL of the freshly prepared suspension of CuBr-DIPEA and azide modified**-**MMs (25 mg, 5 mg/mL). The reaction mixture was shocked at room temperaturewith exclusion of light for 24 h. Then the UA-modified functionalized MMs were separated with magnet and washed three times each with methanol and water. The gathered UA-modified functionalized MMs **(Compound 6)** was used directly for next steps.

**Compound 7**

To a solution of UA-modified functionalized MMs, **Compound 6** (25 mg, 5 mg/mL) in 1 mL methanol, DTT (100 mmol/L) were added. The mixture was shocked at room temperature for 30 min. Then the MMs were separated with magnet and gathered the solution. UA probe released by DTT reduction from above solution was analyzed. LC-MS (ESI): m/z [M+H] calculated for C_39_H_56_N_4_O_2_S: 644; found: 645(Figure S4). The results of LC-MS demonstrated that UA was successfully modified on the surfaces of MMs .

**Figure S4.** LC-MS analysis of the solution of UA-modified functionalized MMs after DTT reduction. (A) The chromatogram and (B) the total ion current of the solution UA-modified functionalized MMs after DTT reduction. (C) The molecular mass of the 7 min chromatography peak.

1. **NF-κB luciferase assay section**

BEAS-2B cells were co-transfected with the NF-κB luciferase reporter plasmid pGL4.32 (100 ng) and Renilla luciferase reporter vector pRL-TK plasmid (9.6 ng). The transfection was performed for 24 h using Lipofectamine 2000 according to the manufacturer’s instructions. Then, the medium was replaced with fresh, serum-free medium 24 h before the experiments. The cells were then pretreated with drugs (UA or alkynyl-modified UA) overnight and stimulated by human TNF-α (20 ng/mL) for 6 h. After stimulation, the cells were washed, lysed, and determined luciferase activity using a dual-luciferase reporter assay system according to the manufacturer’s instructions. The relative luciferase activity was test by normalizing the firefly luciferase activity against the activity of the internal Renilla luciferase control (Modulus™, Turner BioSystems, USA).

As shown in Figure S5, the expression of NF-κB in the Mod group was significantly upregulated after TNF-α stimulation (20 ng/mL). In comparison to the Mod group, pretreatment with Dex significantly inhibited the expression of NF-κB (p< 0.01). Low doses of UA and alkynyl-modified UA (10^-8^mol/L) decreased the production of NF-κB (p< 0.05). Notably, alkynyl-modified UA and UA were equally effective in inhibiting the expression of NF-κB at high or medium doses to varying degrees (10^-6^ mol/L and 10^-7^ mol/L; p<0.01). Therefore, UA and alkynyl-modified UA markedly inhibited the expression of NF-κB dose-dependently in TNF-α-stimulated BEAS-2B cells. It indicated that UA and alkynyl-modified UA exerted the similar anti-inflammation effect.


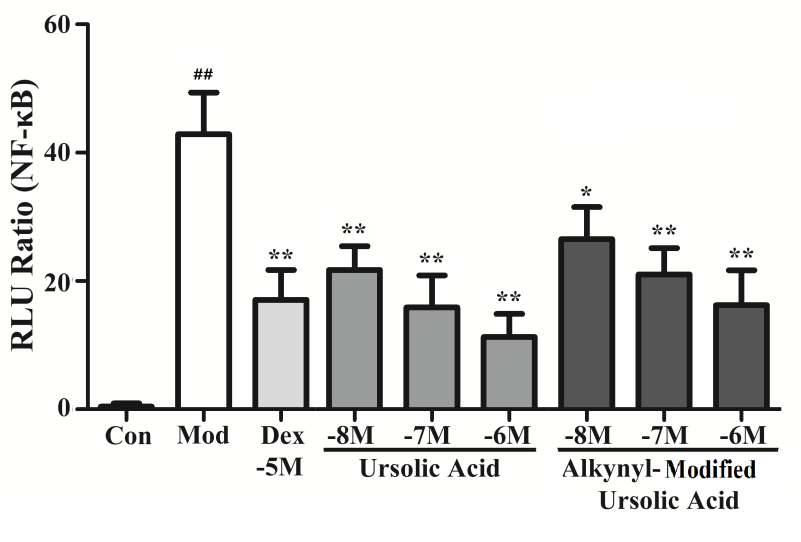


**Figure S5** Inhibitory effects of UA and alkynyl-modified UA on the expression of NF-κB in TNF-α-stimulated BEAS-2B cells. Values are presented as the mean ±SEM, ^##^*p*< 0.01 vs. Con group;**p*< 0.05, ***p*< 0.01 vs. Mod group (n = 5).
